# Mini-Endoscopic Combined Intrarenal Surgery in Patients with Poor Performance Status: A Retrospective Analysis of Postoperative Fever in Over 1000 Cases

**DOI:** 10.5152/tud.2025.25013

**Published:** 2025-06-24

**Authors:** Tadashi Tabei, Hiroki Ito, Takaaki Inoue, Takahiko Watanab, Tetsuo Fukuda, Fukashi Yamamichi, Yosuke Shibata, Junichi Matsuzaki, Kazuki Kobayashi

**Affiliations:** 1 Department of Urology, Fujisawa Shounandai Hospital, Fujisawa, Japan; 2 Department of Urology, Yokohama City University Graduate School of Medicine, Yokohama, Japan; 3 Department of Urology, Hara Genitourinary Hospital, Kobe, Japan; 4 Department of Urology, Ohguchi Higashi General Hospital, Yokohama, Japan; 5 Department of Urology, Yokosuka Kyosai Hospital, Yokosuka, Japan

**Keywords:** Complication, endoscopic combined intrarenal surgery, percutaneous endoscopy, performance status, urolithiasis

## Abstract

**Objective:**

To assess the safety of mini-endoscopic combined intrarenal surgery (mini-ECIRS) in patients with a poor performance status (PS).

**Methods:**

A retrospective analysis was conducted on 1132 patients who underwent mini-ECIRS at 3 hospitals between January 2015 and December 2021. Patients were classified according to their PS (PS0-1 and PS2-4 groups) and compared between the groups in terms of preoperative drainage status, such as ureteral stent or percutaneous nephrostomy (PNS), stone characteristics, surgical outcomes, and postoperative fever. Multivariate logistic regression models were used to identify the predictive factors for postoperative fever in each PS group.

**Results:**

Patients in the PS2-4 group were older and had a higher stone burden than those in the PS0-1 group. The stone-free rates and surgical success rate were similar between the PS groups, but PS2-4 patients had higher rates of postoperative fever without preoperative drainage. Stone composition analysis revealed a higher prevalence of infectious stones in the PS2-4 group. In the PS0-1 group, PNS reduced postoperative fever risk (odds ratio (OR): 0.65, 95% CI: 0.48-0.89, *P* = .01), and history of febrile urinary tract infection, stone burden ≥ 30 mm, number of involved calyces ≥ 4, and female sex were independent risk factors. Notably, in the PS2-4 group, PNS remained effective against postoperative fever (OR: 0.24, 95% CI: 0.07-0.80, *P* = .02), while no other factors were significant.

**Conclusion:**

The mini-ECIRS was effective even in PS-poor patients, and they may benefit more from preoperative PNS placement than normal PS cases.

**Main Points**

- Efficacy and Safety of Mini-Endoscopic Combined Intrarenal Surgery (Mini-ECIRS) in Performance Status (PS)-Poor Patients: The mini-ECIRS demonstrated similar stone-free rates and surgical success between patients with poor PS2-4 and those with normal PS (PS0-1), confirming its safety and efficacy in PS-poor cases.
- Role of Preoperative Percutaneous Nephrostomy (PNS): Preoperative PNS significantly reduced the risk of postoperative fever in both PS groups, with a stronger effect observed in PS-poor patients (odds ratio (OR): 0.24 in PS2-4 vs. OR: 0.65 in PS0-1).
- Distinct Risk Factors for Postoperative Fever: While risk factors like febrile urinary tract infection, stone burden ≥ 30 mm, number of involved calyces ≥ 4, and female sex were identified in the PS0-1 group, these were not significant in the PS2-4 group, highlighting a unique benefit of PNS in mitigating fever risks for PS-poor patients.

## Introduction

Percutaneous nephrolithotomy (PCNL) is the most effective approach for treating renal stones measuring >20 mm. While it is highly effective, it can sometimes lead to serious complications.^1^ Endoscopic combined intrarenal surgery (ECIRS), a technique that combines ureteroscopy and PCNL, has been reported to achieve a higher stone-free rate (SFR) and improved safety.^2^^,^^3^ The recent introduction of mini-ECIRS, which involves the use of smaller endoscope, has further reduced the occurrence of complications.^4^ However, performing surgery on patients with a high perioperative risk remains highly invasive and requires utmost caution. Patients with multiple comorbidities often have asymptomatic stones, and may opt for conservative management. However, certain situations, such as obstructive pyelonephritis, may necessitate active stone removal, posing significant challenges. Although ECIRS is an effective approach for the treatment of large or complex stones,^5^ limited information is available regarding the safety of mini-ECIRS in patients with poor performance status (PS). The group has established the largest database derived from 3 high-volume ECIRS facilities in Japan.^6^^,^^7^ The safety of mini-ECIRS in patients with poor PS is analyzed based on this database

## Material and Methods

### Patients

The clinical criteria for treating urinary stones at the 3 institutions are as follows: for urinary stones less than 20 mm in diameter, shock wave lithotripsy or flexible ureteroscopy is typically recommended. For renal stones larger than 20 mm, mini-ECIRS is offered as the first-line treatment, with flexible ureteroscopy as the second option. The final treatment choice is made after thorough counseling with the patient.

From January 2015 to December 2021, 1417 consecutive patients underwent ECIRS at 3 hospitals. After excluding patients with the intended multistage procedures, 1132 patients were retrospectively analyzed.

Written informed consent was obtained from all the patients for the use of their data for research purposes. The ethics committees of each hospital approved this study and adhered to the principles of the Declaration of Helsinki (Ohguchi East General Hospital: approval number 202201, approved on January 30, 2022; Hara Genitourinary Hospital: approval number 202212021, approved on December 2, 2022; Yokosuka Kyosai Hospital: approval number 20-90, approved on January 20, 2020.

### Data Collection

By reviewing medical records, preoperative information on patient age, sex, Eastern Cooperative Oncology Group Performance Status,^8^ number of stones, stone burden, number of involved calyces, presence or absence of calyceal stones, hydronephrosis, preoperative ureteral stent, percutaneous nephrostomy (PNS), and history of febrile urinary tract infection (f-UTI) were collected. All patients were classified according to their PS: normal PS group (PS0-1) and poor PS group (PS2-4). They were also classified according to the preoperative drainage status (PDS), namely those for whom no drainage had been performed (group: none), only a ureteral stent had been inserted (group: stent), and PNS with or without ureteral stent placement (group: PNS). The stone burden was defined as the sum of the major diameters (mm) of the stones. The results of the stone compositional analysis were classified into infectious or non-infectious groups (infectious: those including struvite or carbonate apatite; non-infectious: others).

### Perioperative Management

Preoperative urine cultures were obtained from all the patients. The patients were administered intravenous antibiotic therapy from the induction of anesthesia until postoperative day 3. If the urine culture result was negative, the patients were given cefmetazole (2 g/day). If positive, antibiotics were administered based on culture results. All surgical procedures were performed under general anesthesia, with the patient in the modified Valdivia position. The details of the surgical technique have been described previously.^7^ In brief, after inserting 14Fr ureteral access sheath, a percutaneous tract with 17.5Fr outer sheath was placed by ultrasonography-guided calyx puncture, followed by one-step dilation. When a PNS had already been placed, the tract was located there. Stone fragmentation was performed using a pneumatic lithotripter or Ho:YAG laser. At the end of the procedure, both a ureteral stent and a PNS tube were placed. The retrieved stone fragments were subjected to compositional analysis.

### Surgical Outcomes

Stone-free status was defined as the absence of any fragments observed on non-contrast computed tomography performed 4 weeks after the operation. Surgical success was defined as having residual fragments less than 4 mm. Cases in which surgery was initiated with the intention of a single session but was conducted in 2 stages for a certain reason (e.g., bleeding) were categorized as non-stone-free. Surgical duration was defined as the entire procedure time (minutes) from the placement of the ureteral access sheath to the urethral or nephrostomy catheter. Postoperative fever was defined as a fever >38°C, which was confirmed by closing the surgery to discharge. Propensity score matching was conducted to assess the risk of postoperative fever while controlling for differences in baseline characteristics between the 2 groups.

In addition to fever, the incidence of major complications such as septic shock, vascular complications, injury to other organs, and transfusion rates was investigated according to PS. Other complications were summarized using the Clavien–Dindo classification, including only those classified as grade II or higher.

### Statistical Analysis

For all statistical tests, statistical significance was set at *P* < .05. Continuous variables are expressed as mean ± standard deviation, whereas categorical variables are expressed as numbers (%). Continuous and categorical variables were compared between the groups using Student’s *t*-test and chi-square test, respectively. A separate multivariate logistic regression model was applied to each PS group to identify risk factors for postoperative fever. This analysis investigate whether these factors—preoperative PNS, ureteral stent, history of f-UTI, stone burden ≥30 mm, number of involved calyces ≥4, female sex, and age—independently affect the occurrence of postoperative fever. The cutoff values of the variants were determined using a receiver operating characteristic curve. All analyses were performed using R, version 4.1.2 (R Foundation for Statistical Computing; Vienna, Austria).

### Data Availability Statement

The datasets analyzed in this study are available from the corresponding author upon request.

## Results

### Patient Background

The number of patients in each PS category was as follows: 1056 in PS0, 38 in PS1, 11 in PS2, 10 in PS3, and 17 in PS4. Table 1 shows the patient background of each PS group. Patients in PS2-4 group are older (57.2 ± 12.9 vs. 62.5 ± 17.4 years, *P* = .01). Stone burden is higher compared to patients in PS0-1 group (32.0 ± 17.7 vs. 47.9 ± 21.5 mm, *P* < .01). They more frequently had a history of f-UTI (14.9% vs. 76.3%, *P* < .01) and some type of preoperative drainage (PDS = none: 66.6% vs. 18.4%, *P* < .01).

### Surgical Outcome and Stone Analysis

Table 2 shows the SFR, surgical success rate (SSR), surgical duration, and postoperative fever of each PDS group comparing between the PS groups. There were no differences in SFR and SSR across any of the PDS. Although not statistically significant, the operative duration was the shortest among patients with PS2-4 with PNS. In the PDS none group, patients in PS2-4 showed much more frequent postoperative fever than those in the PS0-1 group (27.3% vs. 85.7%, *P* < .01). The frequency of postoperative fever improved to the same level as that in the PS0-1 group when the patients underwent any preoperative drainage. Table 3 presents the results of propensity score matching. After balancing baseline characteristics between the 2 groups, no significant difference in the risk of postoperative fever was observed (41.2% vs 35.3%, *P* = .80).

Postoperative complications other than fever stratified by PS are presented in Table 4. All vascular complications were managed with interventional radiology. Pleural injuries were managed by chest tube placement. Renal tract injuries resolved with conservative management. No mortality was observed. Other complications included bladder tamponade, diarrhea, and pneumonia.

The results of stone composition analysis are also presented in Table 2. Approximately half of the stones in the PS2-4 group were infectious, which was significantly higher than those in the PS0-1 group (8.0% vs. 53.6%, *P* < .01).

### Multivariate Analysis for Postoperative Fever

**Figure 1.**
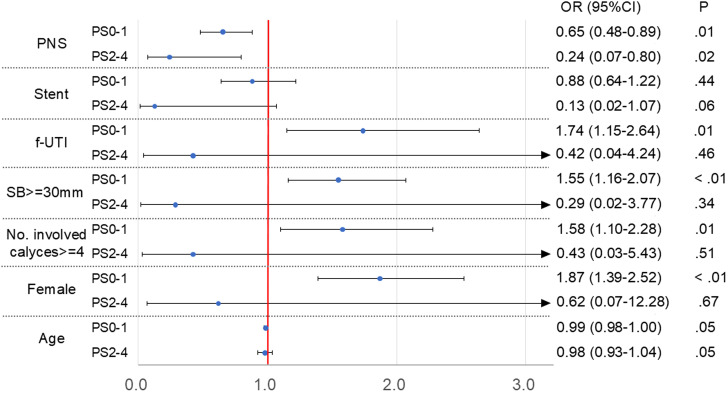
The results of logistic regression analysis for postoperative fever in each PS group. 95% CI; f-UTI, febrile urinary tract infection; OR, odds ratio; PNS, percutaneous nephrostomy; PS, performance status; SB, stone burden.

Figure 1 shows the results of the logistic regression analysis. In the PS0-1 group, the presence of PNS reduced the risk of postoperative fever (OR: 0.65, 95% CI: 0.48-0.89, *P* = .01), while a history of f-UTI (OR: 1.74, 95% CI: 1.15-2.64, *P* = .01), SB ≥ 30 mm (OR: 1.55, 95% CI: 1.16-2.07, *P* < .01), number of involved calyces ≥ 4 (OR: 1.58, 95% CI: 1.10-2.28, *P* = .01), and female sex (OR: 1.87, 95% CI: 1.39-2.52, *P* < .01) were independent risk factors. Interestingly, in the PS2-4 group, the effect of these risk factors was reduced; however, PNS still emerged as a robust countermeasure against postoperative fever (OR: 0.24, 95% CI: 0.07-0.80, *P* = .02).

## Discussion

In this retrospective study, several differences were identified in the backgrounds of patients with poor and normal PS. Patients in the PS2-4 group had a greater stone burden and were more likely to have a history of f-UTI. When surgery was performed without prior drainage, postoperative fever occurred significantly more frequently in PS-poor patients. However, as indicated by the multivariate analysis, PNS emerged as a highly effective preventive measure, mitigating numerous fever risks in PS-poor patients.

According to epidemiological surveys conducted in Japan every 10 years, the incidence of upper urinary tract stones in individuals aged 80 years and older is increasing.^9^ With this trend, the frequency of active stone removal in PS-poor patients is also expected to rise.

Patients with poor PS are susceptible to stone formation due to factors like osteoporosis, urinary stasis, and reduced mobility. Their limited activity makes it challenging for them to naturally expel stones. Even when asymptomatic stones are incidentally discovered, surgical intervention is often deferred due to multiple comorbidities. During this delay, urinary tract infections may develop and sometimes progress to obstructive pyelonephritis.^10^ Only at this stage is active stone removal considered, but as this study shows, the stones typically become larger over time. In fact, 1 study reported that stones in elderly individuals tend to be larger than those in younger patients.^11^

Several studies have reported the safety of active stone removal in patients over 80 years of age.^12^^,^^13^ Although limited research focuses specifically on PS, in a rapidly aging population like Japan, patients over 80 years old with multiple comorbidities are often encountered who still maintain an active daily life, as well as those who spend the majority of their time in bed. To differentiate these patients, this study focused on PS rather than age or comorbidities. One study compared patients with PS3 or higher who underwent active stone removal to those who received conservative treatment. This study not only demonstrated the safety of active stone removal in PS-poor patients but also showed a significant difference in the 2-year stone-specific survival rate (active stone removal: 100% vs. conservative treatment: 61.3%).^14^ These findings support the notion that stones can be life-threatening in elderly and PS-poor patients, and that those eligible for active stone removal should undergo the procedure.

Percutaneous nephrolithotomy is considered a relatively invasive procedure for stone surgery. However, some studies have reported no significant differences in surgical outcomes or complication rates in elderly patients.^15^^,^^16^ Data from the Clinical Research Office of the Endourological Society showed that, while the SFR remained consistent in patients aged ≥ 70 years, the incidence of complications was higher in this age group.^17^ Among patients ≥ 70 years, the American Society of Anesthesiologists (ASA) III has been identified as a risk factor for complications.^18^ Although parameters such as age, PS, and ASA score are complex to interpret individually, endourologists should evaluate PCNL feasibility by considering a comprehensive view of comorbidities and PS, rather than relying solely on age. Schulz proposed an algorithm to approach the surgical management in older patients based on their thorough review of previous studies on this subject. They proposed considering comorbidities, functional status, and patient priorities.^19^

To the best of knowledge, research specifically focusing on PS-poor cases in ECIRS has not been reported. A previous study identified several factors predicting the occurrence of systemic inflammatory response syndrome after ECIRS, including the number of involved calyces, stone surface area, and a history of f-UTI. Although PS was among the factors analyzed, the number of PS-poor patients was limited, resulting in insufficient data to draw meaningful conclusions for this population.^20^ Many previous studies, as noted, have shown that poor PS or older age can be risk factors for complications in PCNL. However, few have compared risk factors for complications between patients with poor PS and those with normal PS to propose appropriate treatment strategies. This study provides meaningful data in this regard, which was made possible by the large sample size available in the research.

Regarding the current study, it is not surprising that the SFR were equivalent; however, it is noteworthy that the surgical duration did not change significantly, despite the PS2-4 group having a greater stone burden. An intriguing aspect of this study was the identification of risk factors for postoperative fever. Stone analysis revealed that cases with an infectious background are common in PS-poor patients, and without preoperative drainage, the likelihood of fever increases significantly. However, this risk can be greatly mitigated by inserting a preoperative ureteral stent or PNS. While the effectiveness of PNS in reducing infectious complications in ECIRS has been reported previously,^21^ the present findings provide novel insight by highlighting its potentially greater benefit in patients with poor PS. The results of the multivariate analysis were particularly revealing. Preoperative PNS placement is considered effective in preventing postoperative fever by stabilizing the internal tract surface and reducing the likelihood of bacteria entering the bloodstream from the urinary tract. The PS-poor patients are more likely to have an infectious background, which may explain their greater benefit from PNS, as reflected in their lower odds ratio for postoperative fever compared to normal PS patients. While PNS act as a preventive factor even in the normal PS group, the prevalence of non-infectious stones, which are typically harder, leads to significantly increased surgical difficulty, especially when stones are located in multiple calyces.^5^ These challenges cannot be resolved solely through the use of PNS. Conversely, in PS-poor cases, even when the stone size is large and stones are located in multiple calyces, they are often softer due to their infectious origin. This characteristic allows for relatively easy expulsion through passive fragment retrieval^22^ or other measures using a flexible ureteroscope, which is typically applied only in the ECIRS procedure and not in PCNL. Although the shorter surgical duration observed in PS-poor patients with PNS was not statistically significant, it can be attributed to these specific stone characteristics.

This study has several limitations. First, because it is a retrospective analysis, various biases must be taken into account. The database consists of patients who underwent surgery, meaning that all patients were in a systemic state capable of tolerating general anesthesia. Consequently, those in poor systemic health who could not endure surgical intervention were not included. Therefore, the feasibility of surgical procedure should be evaluated on an individual basis. Second, it is important to acknowledge that these data are derived from high-volume facilities where ECIRS is performed regularly, and technical issues are minimal. The applicability of these results may be limited in environments with less experience or more complications. Third, information regarding recurrence rates was not available in the database. A high recurrence rate is expected among patients with poor PS. Therefore, careful consideration of surgical indications is necessary, alongside a thorough discussion of surgical risks and long-term prognosis. Fourth, due to the relatively small number of cases in the PS-poor group, the statistical power may be limited. While increasing the sample size could yield different outcomes, the current analysis does not appear to contradict the experiential evidence of experts in the field. To overcome these limitations, a larger randomized prospective study involving multiple institutions is needed. Nonetheless, a reasonable solution for treating PS-poor patients is presented based on an extensive database of mini-ECIRS.

In conclusion, mini-ECIRS was effective even in PS-poor patients, and they may benefit more from preoperative PNS placement than normal PS cases.

## Figures and Tables

**Table 1.** Patients’ Characteristics

Variants		PS0-1	PS2-4	*P*
	Unit/Category	Mean ± SD/no (%)		
n	-	1094	38	-
Age	Years	57.2 ± 12.9	62.5 ± 17.4	.01
Sex	Female	349 (31.9)	18 (47.3)	.05
Stone burden	mm	32.0 ± 17.7	47.9 ± 21.5	<.01
No. of stones	≤5	953 (77.2)	35 (85.4)	.25
No. of involved calyces	≤3	924 (84.5)	30 (78.9)	.36
Calyceal stone	Present	892 (81.5)	35 (92.1)	.13
Hydronephrosis	Present	719 (65.7)	21 (55.3)	.22
History of f-UTI	Present	163 (14.9)	29 (76.3)	<.01
PDS	None	729 (66.6)	7 (18.4)	<.01
	Stent	270 (24.7)	11 (28.9)	
	PNS (±stent)	95 (8.7)	20 (52.6)	

BMI, body mass index; f-TUI, febrile urinary tract infection; No., number; PDS, preoperative drainage status; PNS, percutaneous nephrostomy; SD, standard deviation.

**Table 2.** Surgical Outcome According to PDS Group and Stone Analysis

	PDS	PS0-1	PS2-4	*P*
SFR	Total	530 (48.4)	19 (50.0)	.98
	None	354 (48.5)	5 (71.4)	.40
	Stent	141 (52.2)	4 (36.3)	.46
	PNS	35 (36.8)	10 (50.0)	.39
SSR	Total	862 (78.8)	34 (89.5)	.16
	None	568 (77.9)	6 (85.7)	.97
	Stent	228 (84.4)	10 (90.9)	.87
	PNS	66 (69.5)	18 (90.0)	.11
Surgical duration (minutes)	Total	110.9 ± 34.1	100.4 ± 27.2	.06
	None	110.9 ± 32.4	106.5 ± 12.5	.71
	Stent	112.1 ± 37.3	106.6 ± 30.6	.63
	PNS	107.3 ± 37.3	94.9 ± 28.8	.16
Postoperative fever	Total	291 (26.7)	12 (31.5)	.63
	None	199 (27.3)	6 (85.7)	<.01
	Stent	76 (28.2)	4 (36.3)	.51
	PNS	16 (17.0)	2 (10.0)	.73
Stone analysis	Non-infectious	1003 (92.0)	18 (47.4)	<.01
	Infectious	87 (8.0)	20 (53.6)	

PDS, preoperative drainage status; PNS, percutaneous nephrostomy; SFR, stone-free rate; SSR, surgical success rare.

**Table 3.** Patient Characteristics and Incidence of Postoperative Fever After Propensity Score Matching

Variants		PS0-1	PS2-4	*P*
	Unit/Category	Mean ± SD/no. (%)		
n	-	34	34	-
Age	Years	61.2 ± 12.2	61.4 ± 17.6	.96
Sex	Female	16 (47.1)	16 (47.1)	.99
Stone burden	mm	40.2 ± 23.2	46.0 ± 21.1	.28
No. of stones	≤5	31 (91.2)	31 (91.2)	.99
No. of involved calyces	≤3	30 (88.2)	26 (76.5)	.34
Calyceal stone	Present	27 (79.4)	31 (91.2)	.30
Hydronephrosis	Present	21 (61.8)	20 (58.8)	.99
History of f-UTI	Present	29 (85.3)	25 (73.5)	.36
Pre-operative drainage	None	6 (17.6)	7 (20.6)	.80
	Sstent	13 (38.2)	11 (32.4)	
	PNS (±stent)	15 (44.1)	16 (47.0)	
Postoperative fever	Present	14 (41.2)	12 (35.3)	.80

BMI, body mass index; f-TUI, febrile urinary tract infection; no., number; PDS, preoperative drainage status; PNS, percutaneous nephrostomy; SD, standard deviation.

**Table 4.** Postoperative Complications Other than Fever

	PS0-1	PS2-4
Septic shock	21 (1.92%)	2 (5.26%)
Vascular complication	5 (0.44%)	0 (0%)
Pleural injury	6 (0.55%)	0 (0%)
Blood transfusion	4 (0.37%)	1 (2.63%)
Renal tract injury	9 (0.83%)	0 (0%)
Death	0 (0%)	0 (0%)
Others		
Bladder tamponade (Grade 2)	5 (0.46%)	0 (0%)
Diarrhea (Grade 2)	0 (0%)	1 (2.63%)
Pneumonia (Grade 2)	1 (0.09%)	0 (0%)

## Data Availability

The data that support the findings of this study are available on request from the corresponding author.
